# Bactericidal activity of alpha-bromocinnamaldehyde against persisters in *Escherichia coli*

**DOI:** 10.1371/journal.pone.0182122

**Published:** 2017-07-27

**Authors:** Qingshan Shen, Wei Zhou, Liangbin Hu, Yonghua Qi, Hongmei Ning, Jian Chen, Haizhen Mo

**Affiliations:** 1 Department of Food Science, Henan Institute of Science and Technology, Xinxiang, China; 2 Department of Animal Science, Henan Institute of Science and Technology, Xinxiang, China; 3 Institute of Food Quality and Safety, Jiangsu Academy of Agricultural Science, Nanjing, China; Institut Pasteur, FRANCE

## Abstract

Persisters are tolerant to multiple antibiotics, and widely distributed in bacteria, fungi, parasites, and even cancerous human cell populations, leading to recurrent infections and relapse after therapy. In this study, we investigated the potential of cinnamaldehyde and its derivatives to eradicate persisters in *Escherichia coli*. The results showed that 200 μg/ml of alpha-bromocinnamaldehyde (Br-CA) was capable of killing all *E*. *coli* cells during the exponential phase. Considering the heterogeneous nature of persisters, multiple types of persisters were induced and exposed to Br-CA. Our results indicated that no cells in the ppGpp-overproducing strain or TisB-overexpressing strain survived the treatment of Br-CA although considerable amounts of persisters to ampicillin (Amp) and ciprofloxacin (Cip) were induced. Chemical induction by carbonyl cyanide m-chlorophenylhydrazone (CCCP) led to the formation of more than 10% persister to Amp and Cip in the entire population, and Br-CA still completely eradicated them. In addition, the cells in the stationary phase, which are usually highly recalcitrant to antibiotics treatment, were also completely eradicated by 400 μg/ml of Br-CA. Further studies showed that neither thiourea (hydroxyl-radical scavenger) nor DPTA (Fe^3+^ chelator to block the hydroxyl-radical) affected the bactericidal efficiency of the Br-CA to kill *E*. *coli*, indicating a ROS-independent bactericidal mechanism. Taken together, we concluded that Br-CA compound has a novel bactericidal mechanism and the potential to mitigate antibiotics resistance crisis.

## Introduction

The current antibiotic resistance crisis arising from the large-scale use of antibiotics has led to increased pressure to prioritize strategies to tackle the issue in clinical practice [1]. The need for new antibiotics is more pressing due to the presence of persister cells, which are defined as a small fraction of a bacterial population displaying non-hereditary tolerance to high doses of bactericidal antibiotics [2, 3]. Persistence has been widely described in bacteria, fungi, parasites, and even cancerous human cell populations, which ultimately led to recurrent infections and relapses after therapy [4]. A plethora of environmental signals such as nutrient limitation, pretreatment by sub-inhibitory concentrations of antibiotics, oxidative stress, heat shock, or DNA-damaging agents, can all increase persister levels, which poses a great challenge to antibiotic therapy as well [5–9].

Ubiquitous bacterial stress alarmone ppGpp is widely accepted as an emerging central regulator of persistence both in stochastically and environmentally induced persistence through its effectors, toxin-antitoxin modules [9]. In addition, induction of the SOS response dramatically increases persistence to fluoroquinolones, which is dependent on the DNA damage-inducible TisB toxin [6]. Both pathways are supported by the fact that rifampicin (Rifa, halting transcription), tetracycline (Tetra, halting translation), and carbonyl cyanide m-chlorophenylhydrazone (CCCP, halting ATP synthesis), which mimic type II and TisB-like toxins, respectively, could dramatically increase persistence [10]. Based on the known persister characteristics, several approaches, e.g., alteration of membranes via Trp/Arg-containing antimicrobial peptides [11], activation of ClpP-mediated self-digestion via Rifa and ADEP4 [12], and conversion of persisters to non-persisters via cis-2-decenoic acid [13] have been proposed. The discovery that some individual compound such as mitomycin C [14], boromycin [15], and brominated furanones [16] had the potential to eradicate persisters is also very promising [11]. Considering the diverse and variable nature of persisters [17], more drugs need to be developed based on the screening of extensive compound resources.

Ancient medical treatments against microbial infection mainly depended on herbal plant resources [18]. In particular, essential oils have frequently been found to display wide-spectrum antimicrobial activity. Cinnamaldehyde (CA), a kind of essential oil, has potent antimicrobial activity against bacteria and fungi [19, 20]. Recently, CA has been indicated to dramatically inhibit the production of biofilm in many microbes [21–23]. It has been generally accepted that persister cells are responsible for the relapse of biofilm infections [24, 25]. Therefore, we hypothesized that novel persister eradicators could be found from CA derivatives. In this study, we determined the activity of CA and its eight derivatives against persisters in *E*. *coli*, and found that alpha-bromocinnamaldehyde (Br-CA) had great potential to eradicate persisters.

## Materials and methods

### Strains, culture conditions, and chemicals

The *E*. *coli* strains used are listed in Table 1. All experiments were conducted at 37°C in Luria-Bertani (LB) medium shaking at 220 rpm (liquid cultures) [26]. Rifa, Tetra, CCCP, ciprofloxacin (Cipro), and ampicillin (Amp) were purchased from Sigma-Aldrich. CA and its derivatives, including α-methylcinnamaldehyde, cinnamic alcohol, ο-nitrocinnamaldehyde, Br-CA, carbicol, phenylpropyl aldehyde, phenylpropanoic acid, and cinnamic acid, were obtained from Tokyo Chemical Co. LTD (TCI). Other reagents were of standard analytical purity.

**Table 1:** Bacterial strains and plasmids used in this study.

Strains and plasmids	Description	Source or reference
*E*. *coli* K-12 Strains		
MG1655	F^-^ λ^-^ *ilvG rfb-50 rph-1*, *recA*	K. Lewis
MG1655 *ΔrecA*	*ΔrecA*::Kan	[6]
**Plasmids**		
pZS*24	Kan^R^; lacl^q^, *E*. *coli* expression vector	[6]
pZS*24*tisB*	Kan^R^; lacl^q^,*tisB*	[6]
pCA24N	Cm^R^; lacl^q^, *E*. *coli* expression vector	[6]
pCA24N*relA*	Cm^R^; lacl^q^, *relA*	[27]

### Persister measurement

*E*. *coli* MG1655 was cultured in LB overnight and then diluted 1:200 into fresh LB medium. The diluted cultures were incubated at 37°C with shaking to reach OD600 = 1.0 or stationary growth phase (OD600 = 3.0). The persister formation was evaluated post exposure to 200 μg/ml of Amp and 5 μg/ml of Cipro for 4 hour as described by Orman [25]. Simultaneously, the cells during these phases were treated with CA and its derivatives and the corresponding persisters levels measured. The final concentrations of CA and its derivatives were 5, 10, 50, 100, 200 and 400 μg/ml. The samples were all incubated at 37°C for 4 hour, and then washed with saline twice. The cell viability was determined using the drop plating method [28]. Spread plating was done to repeat all of the experiments. The assay was repeated at least three times. The hierarchical cluster analysis was performed based on their dose-dependent bactericidal activities using Cluster 3.0 software in the CLUSTER program (http://bonsai.hgc.jp/~mdehoon/software/cluster/software.htm), and the resulting tree figures and cluster color bar were displayed using Java Treeview (http://www.treeview.net/) [29].

### Measurement of persisters induced by TisB and RelA overexpression

Overexpression of RelA and TisB in strains MG1655/pCA24N*relA* and MG1655/pZS*24*tisB*, respectively, was used to induce persister formation. Briefly, overnight cultures were inoculated at a ratio of 1:200 into fresh LB. Chloramphenicol (30 μg/ml) and kanamycin (50 μg/ml) were used for maintaining the plasmids pCA24N*relA* and pZS*24*tisB*, respectively. When OD600 value reached 0.4, the culture was supplemented with Isopropyl-β-d-thiogalactoside (IPTG) (0.3 mM for pCA24N*relA* and 0.5 mM for pZS*24*tisB*) to induce *relA* and *tisB* overexpression. The cultures were continually incubated until the OD600 value approached 1.0. The cells were collected through centrifugation at 5,000 g for 5 min. The culture medium was discarded and the cells were resuspended in LB medium containing 100 μg/ml of Amp, 5 μg/ml of Cipro and 200 μg/ml of Br-CA respectively, and incubated for 4 hour. Simultaneously, MG1655 containing empty plasmid was included as a control and was treated under the same conditions. Cell viability was determined using the drop plating method [28].

### Measurement of persisters induced by chemicals

As described above [10], culture of MG 1655 was grown until OD600~1.0. Then, the cultures were pretreated with Rifa (100 μg/ml for 30 min), Tetra (50 μg/ml for 30 min), or CCCP (20 μg/ml for 3 hour). Following the pretreatment, the cultures were centrifuged to remove the pretreatment compounds and resuspended in fresh LB containing ciprofloxacin (5 μg/ml), ampicillin (100 μg/ml), and Br-CA (200 μg/ml) respectively before being incubated for 4 hour [10]. Cell viability was determined using the drop plating method [28].

### MIC and MBC Assays

The minimum inhibitory concentration (MIC) of Br-CA against *E*. *coli* MG1655 was determined through broth micro dilution assay in a 96-well plate. The bacterial culture during the mid-late exponential phase was prepared and diluted with fresh LB medium until the OD600 value reached 0.05. Subsequently, the diluted culture was transferred into a 96-well plate with 200 μl in each well. Br-CA was added into the wells with final concentrations of 0, 20, 40, 80 and 100 μg/ml. After incubation for 24 hour at 37°C, OD600 value in each treated well was determined using a microplate reader (Thermo Fischer, United Kingdom). The MIC was defined as the minimum concentration of Br-CA leading to no significant increase of OD600 value. At the same time, the cell viability in each well was assessed using the drop plating method after being washed with saline [28]. The minimum bactericidal concentration (MBC) of Br-CA was defined as the minimum concentration of Br-CA leading to no viable cell determined in the well.

### Hydroxyl-radical scavenging

According to Kohanski [30] and Piccaro [31], thiourea (Thio) was used as a hydroxyl-radical scavenger and diethylene triaminepenta acetic acid (DTPA) was used as a Fe^3+^ chelating agent to block the hydroxyl-radical production. Thio (final concentration of 100 mM) or DTPA (final concentration of 1 mM) was added into the culture 3 min before the exposure to Br-CA. The effects of Thio and DTPA on the bactericidal activity of Br-CA were assessed in the case of 200 μg/ml of Br-CA against the cells of OD600 = 1.0 or Br-CA at MIC and MBC, respectively against the cells of OD600 = 0.05.

### Data analysis

All of the experiments in this study were repeated three times, and the results shown are the mean ± SE of the three independent experiments. Significant differences between the treatments were evaluated using SD and one-way analysis of the variance (ANOVA) using SPSS 2.0. The data between the two specific different treatments were compared statistically using Student’s *t*-test. The differences were considered significant at *P*<0.05.

## Results

### Bactericidal activities of CA and its derivatives

**Fig 1:**
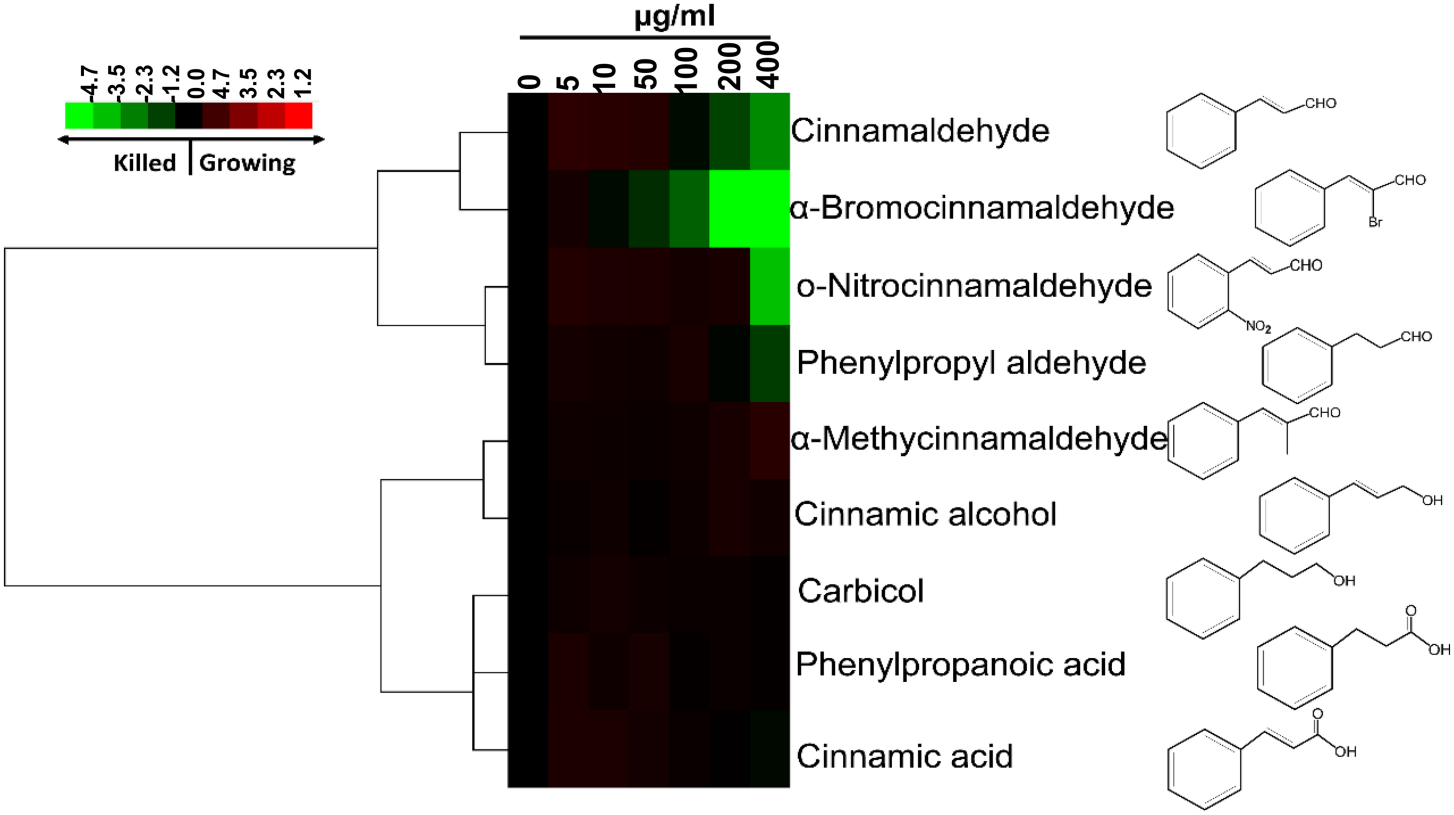
Hierarchical cluster analysis of the bactericidal effects of CA and its derivatives on *E*. *coli* cells in exponential growth phase. Cell survival percentage after treatment was calculated based on the colony counts on the LB agar plates. Resulting tree figure was obtained using Cluster 3.0 software and Java Treeview. The cluster color bar was shown as [log (cell survival percentage ×100)-2]. If the given amount of drug failed to completely inhibit the bacterial growth, the corresponding values in the bar would be positive in red color; If the given amount of drug showed the bactericidal effects, the corresponding values in the bar would be negative in green color.

The number of persisters increases during the mid-exponential phase [32]. We assessed the bactericidal effects of CA and the eight CA derivatives, i.e. Br-CA, α-methylcinnamaldehyde, cinnamic alcohol, ο-nitrocinnamaldehyde, carbicol, phenylpropyl aldehyde, phenylpropanoic acid, and cinnamic acid on *E*. *coli* MG1655 cells during the exponential phase. As shown in Fig 1, only CA (killing rate: 99.8%), Br-CA (killing rate: 100%), ο-nitrocinnamaldehyde (killing rate: 99.8%), and phenylpropyl aldehyde (killing rate: 92.1%) showed significant bactericidal effects at the given concentrations. Through cluster analysis based on their dose-dependent activity, these four compounds were also distributed into the same subgroup, and all of them have aldehyde group (Fig 1). Among these derivatives, Br-CA exhibited the most effective bactericidal activity. When the concentration of Br-CA reached 100 μg/ml, less than 1% of cells survived. Treatment with Br-CA at concentrations over 200 μg/ml completely killed all cells including persisters to common antibiotics.

### MIC and MBC of Br-CA against *E*. *coli* MG1655

**Fig 2:**
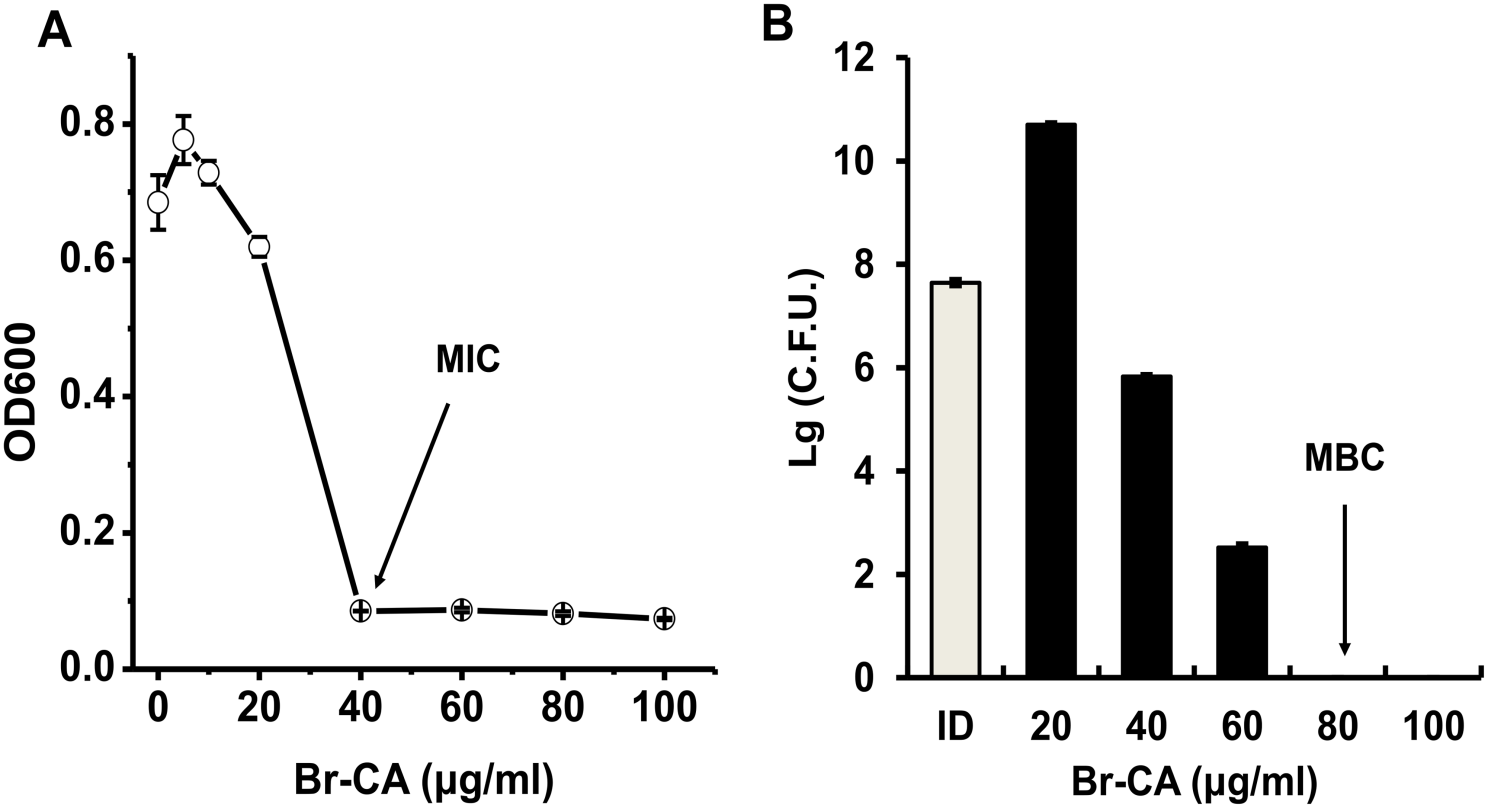
Determination of MIC (A) and MBC (B) of Br-CA against *E*. *coli*. MIC was defined as the lowest concentration of Br-CA to completely inhibit cell growth at 37°C in 96 wells plates for 24 hour and OD600 was monitored with the microplate reader to indicate the cell growth. MBC was defined as the lowest concentration of Br-CA to completely eradicate all the cells incubated at 37°C in 96 wells plates for 24 hour and surviving cells were evaluated using drop plating method after serial dilutions. ID stood for the initial cell density. Each data point or bar was indicated as the means of three replicates ± standard deviation.

The MIC value of Br-CA against *E*. *coli* MG1655 was obtained through a micro-well culture plate assay using serial dilutions of Br-CA. The MBC value was determined by determining the viability of cells in the micro-wells. As shown in Fig 2A and 2B, the MIC value and MBC value of Br-CA against *E*. *coli* MG1655 were 40 μg/ml and 80 μg/ml, respectively. Br-CA was regarded as bactericidal because the MBC is no more than four times of the MIC according to French [33]. Based on the MIC and MBC of Br-CA, 200 μg/ml of Br-CA (concentration of 5×MIC) was chosen to investigate its potential to eradicate the persister cells in *E*. *coli* MG1655.

### Br-CA eradicated persisters in stationary phase

**Fig 3:**
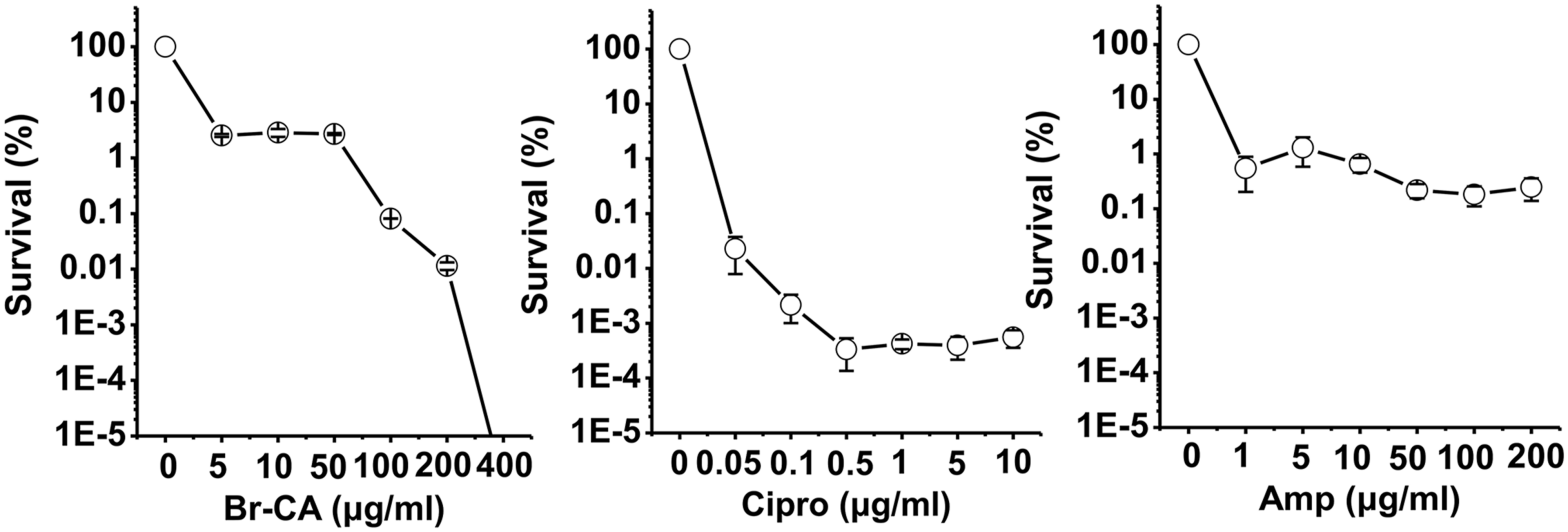
Killing curves of MG1655 cells in the stationary phase by Br-CA, Cipro and Amp. Cell survival percentage after treatment was calculated based on the colony counts on the LB agar plates. Each data point or bar was indicated as the means of three replicates ± standard deviation.

It has been well accepted that more persisters are formed during the stationary phase [34], which was supported by our results that in stationary phase treated by Amp the level of persisters increased to approximately 1% of the population, while it remained at low level when cells were treated by Cipro (Fig 3). Br-CA displayed a different dose-dependent activity against stationary phase *E*. *coli* cells as compared to exponential phase cells, but it was able to eradicate the stationary phase persisters when its concentration reached 400 μg/ml.

### Br-CA eradicated persisters induced by toxin overexpression

**Fig 4:**
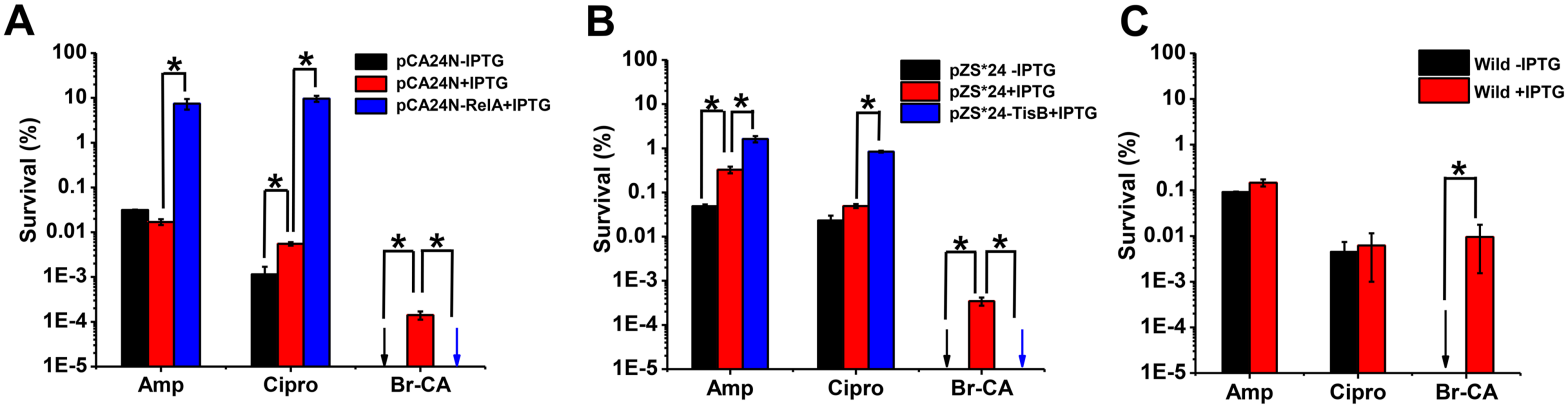
The persisters induced by toxin overexpression were eradicated by Br-CA. **(A)** The persisters were induced by RelA overexpression and the effects of IPTG on the persister formation in MG1655/pCA24N; **(B)** The persisters were induced by TisB overexpression and the effects of IPTG on the persister formation in MG1655/pZS*24; **(C)** The effects of IPTG on the persister formation in MG1655. Cell survival percentage after treatment was calculated based on the colony counts on the LB agar plates. The arrows indicated that persister cells were eradicated completely and different colors showed different treatments. Each data point or bar was indicated as the means of three replicates ± standard deviation. Asterisks indicate a significant difference from their controls (*P* <0.05).

Since ppGpp can induce the formation of persisters [9], we employed the RelA overexpression strain MG1655/pCA24N*relA* in which ppGpp was over produced to induce persister formation. Our results showed that overproduction of ppGpp induced more than 430-fold and 1890-fold of persisters induced to Amp and Cipro, respectively (Fig 4A). Br-CA completely eradicated all of the cells (killing rate: 100%) including persisters induced by ppGpp. The toxin TisB in SOS can also induce persister formation through the elimination of cellular ATP [6]. In the present study, the MG1655/pZS*24*tisB* strain in which TisB was overexpressed led to 4.9-fold and 17-fold increases in persister formation as compared to those induced by Amp and Cipro, respectively (Fig 4B). Similar to its efficiency against the strain pCA24N*relA*, Br-CA also eradicated all pZS*24 *tisB* cells (killing rate: 100%). Surprisingly, a small fraction of cells containing empty vectors survived Br-CA treatment in the presence of IPTG (Fig 4A and 4B), indicating that IPTG probably helped cell survival after exposure to Br-CA. Indeed, in absence of IPTG, persisters were eradicated by Br-CA while only slight changes occurred in persisters levels obtained after treatment by Amp and Cipro. In addition, we also determined the effects of IPTG on persister formation in wild type strains. In comparison to the control group (without IPTG), addition of IPTG did not significantly change the levels of persisters when treated by Amp and Cipro, whereas it substantially increased persisters levels when treated by Br-CA (Fig 4C).

### Potential of Br-CA against chemical-induced persister formation

**Fig 5:**
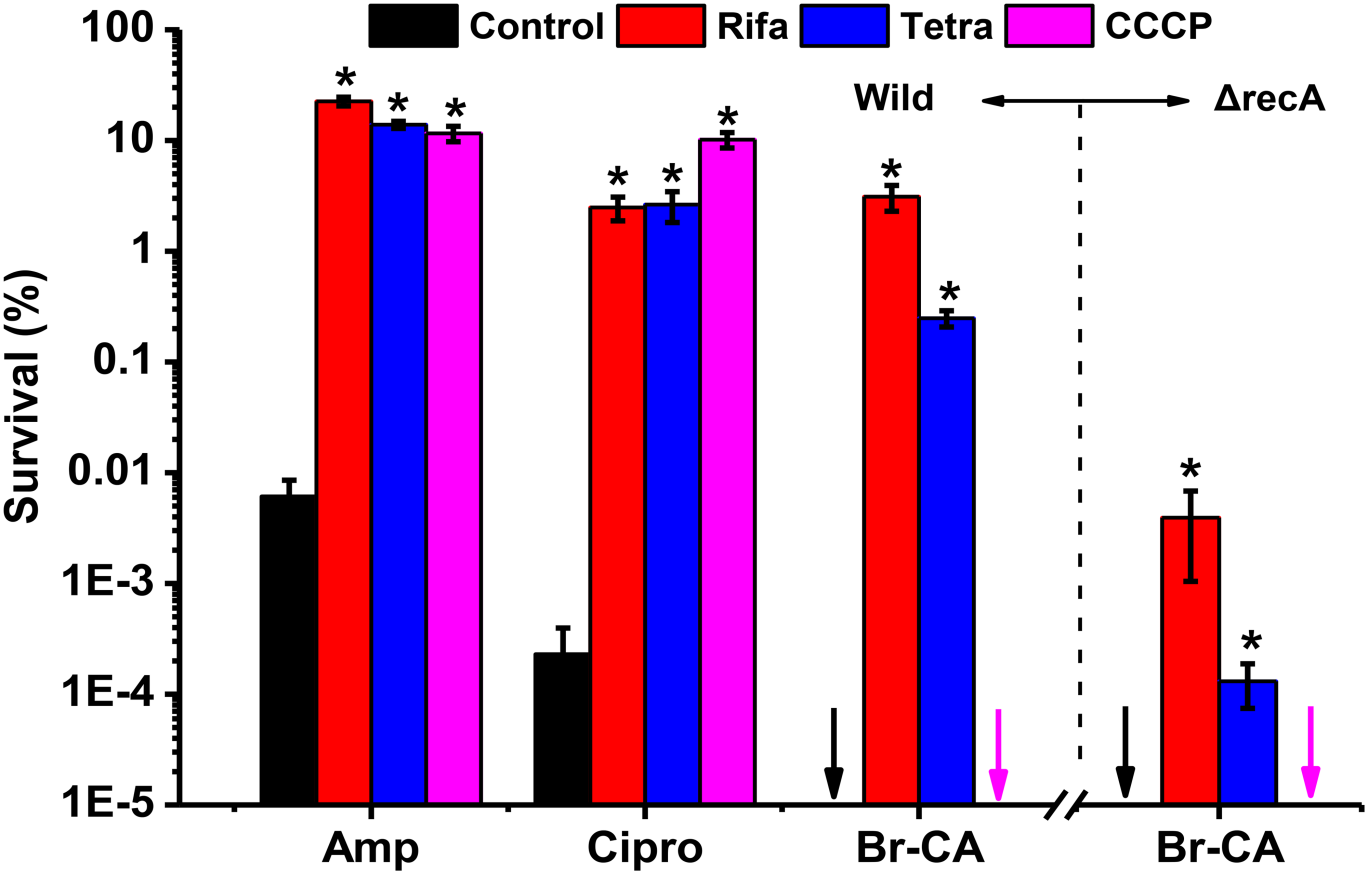
Persister cells induced by chemicals were challenged by Br-CA. Cells were pretreated with Rifa, Tetra or CCCP, respectively except the control group and then exposed to Cipro, Amp and Br-CA for 4 hour. Cell survival percentage after treatment was calculated based on the colony counts on the LB agar plates. The arrows indicated that persister cells were eradicated completely and different colors showed different treatments. Results are means from three independent experiments ± standard deviation. Each bar was indicated as the means of three replicates ± standard deviation. Asterisks indicate a significant difference from their controls (*P* <0.05).

Rifampin (halting transcription), tetracycline (halting translation), and CCCP (halting ATP synthesis) have been used to mimic type II and TisB-like toxins to induce persister formation, respectively [10]. In our current study, these three compounds all led to more than 1000-fold increase in the persister levels when treated by Amp or Cipro (Fig 5). Br-CA completely eradicated all cells (killing rate, 100%) under induction by CCCP (Fig 5), which was in line with the results from the overexpression of TisB (Fig 4B). However, Br-CA failed to eradicate the cells under the induction of Rifa and Tetra (Fig 5), and it was obvious that persisters induced by Rifa or Tetra were also tolerant to Br-CA (Fig 5). This is inconsistent with the results from the overproduction of ppGpp, indicating that the mechanism underlying persister formation by Rifa and Tetra was different from the ppGpp pathway. In strain *ΔrecA* (a mutant with the deletion of *recA* to block SOS), persisters levels after treatment by Br-CA and pre-treatment by Rifa and Tetra decreased by 790-fold and 1906-fold, respectively, as compared to the wild type (Fig 5).

### The role of ROS in the bactericidal activity of Br-CA

**Fig 6:**
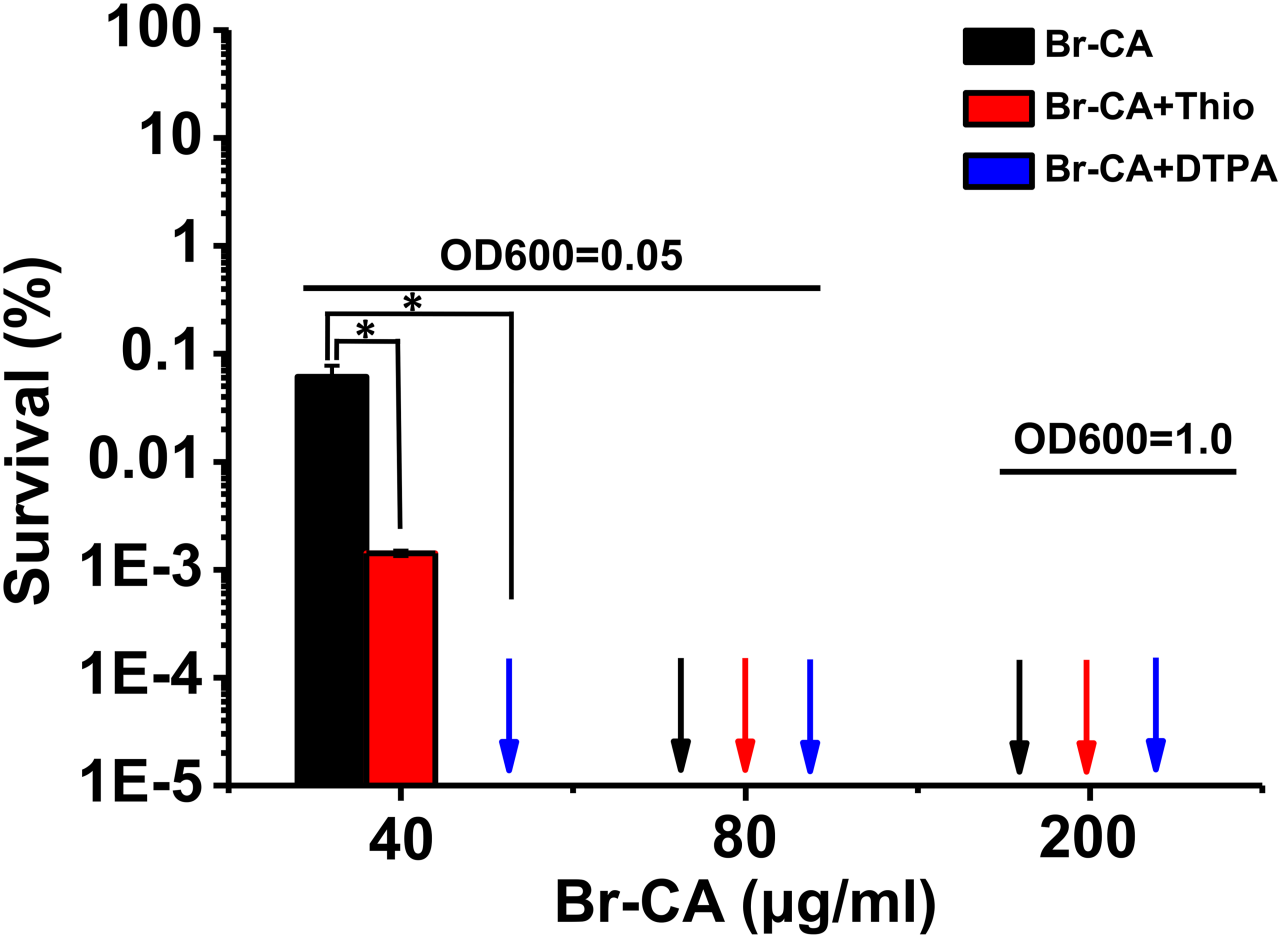
Thiourea and DTPA have no effect on the eradication of persisters by Br-CA. The initial bacterial suspension (OD600 = 0.05 or OD600 = 1.0) was pretreated with or without Thio or DTPA iron chelator and then exposed to different concentrations Br-CA. Cell survival percentage after treatment was calculated based on the colony counts on the LB agar plates. The arrows indicated that cells were killed completely by Br-CA and different colors showed different treatments. Each bar was indicated as the means of three replicates ± standard deviation. Survival post treatment with Br-CA alone at the given concentration were set as the control in the corresponding group. Asterisks indicate a significant difference from their controls (*P* <0.05).

Reactive oxygen species (ROS) are known to cause important damage to the cell, which can lead to cell death [35, 36]. In order to investigate whether ROS was involved in the high bactericidal activity of Br-CA, thiourea (hydroxyl-radical scavenger) and the iron chelator DTPA (blocking hydroxyl-radical production) were employed. As shown in Fig 6, the addition of Thio or DTPA either did not affect the eradication of exponential cells of *E*. *coli* under Br-CA treatment. Further, the effects of Thio or DTPA on the bactericidal activity of Br-CA at MBC (80 μg/ml) and MIC (40 μg/ml) was also investigated, respectively. The results showed that Thio or DTPA did not antagonize the bactericidal effects of Br-CA at MBC (80 μg/ml), and they even promoted bacterial death by Br-CA at MIC (40 μg/ml) (Fig 6). These results suggested that ROS could play a protective role rather than causing cell death in *E*. *coli* after exposure to Br-CA.

## Discussion

As a derivative of the essential oil CA, Br-CA is capable of causing slightly non specific inflammatory irritating effects on the skin and eye [37]. According to the safety data sheet of the products by Sigma-Aldrich Co., Br-CA is defined as chemical of the 4^th^ grade of acute toxicity in oral administration (LD50> = 2000 mg/Kg). Br-CA has been reported to possess a dose-independent genotoxic and mutagenic potential in bacteria [38], but to date, no DNA-adducts of Br-CA or any clear carcinogenic activities have been found. The present study demonstrated Br-CA had the most powerful bactericidal activity against *E*. *coli* among CA and its derivatives, with especially high efficiency in the eradication of persisters. Balaban and colleagues [39] have defined two types of *E*. *coli* persisters based on their growth-rate and formation period, i.e. type I persisters forming from the stationary phase and type II persisters that are continuously generated during growth. Type I persisters especially reach 1% of the entire population [34]. It was inspiring that Br-CA could eradicate both type I and type II persisters. Recent studies have indicated that the anticancer drugs mitomycin C and cisplatin eradicate persisters by cross-linking their DNA [14, 40]. However, this kind of bactericidal mechanism also leads to significant side effects [41, 42], and they are also very costly. In comparison to them, Br-CA has a low price, showing potential to be applied in the treatment of infection. However, there is very scarce data regarding Br-CA toxicity, and we should be cautious in its application in the treatment of infections.

The ppGpp has been recognized as a central regulator that inhibits translation and cell growth, thereby inducing persistence through activation of toxins [9]. Our results showed that Br-CA completely eradicated the persisters induced by the overproduction of ppGpp, but not that induced by Rifa or Tetra. Since the antimicrobial roles of Rifa and Tetra have been targeted at the RNA polymerase and 30S ribosomal subunit, respectively [43, 44], we proposed that ppGpp-induced persister formation is independent of the inhibition of transcription or translation. The recent study has shown that ppGpp couples transcription to DNA repair in *E*. *coli*, which contributes to its survival under genotoxic stress [45]. Therefore, SOS may be involved in ppGpp-induced persister formation to some antibiotics. Blocking SOS by deleting recA significantly decreased Rifa- or Tetra-induced persisters to Br-CA as compared to the wild-type (Fig 5), which suggests the involvement of SOS in the induction of persister formation by Rifa or Tetra. Dörr et. al., [6] have identified a SOS-associated persister formation pathway through the activation of TisB, leading to ATP depletion. However, Br-CA effectively eradicated the persisters induced by either TisB overexpression or CCCP. This suggested that there might be other elements in SOS in addition to TisB that induce the persister formation. IPTG increased the cells that survived the Br-CA treatment, whereas the overexpression of toxins eliminated this protective effect of IPTG, indicating that toxins overexpression leading to persister formation to Amp and Cipro could be helpful in the eradication of persisters by Br-CA. Taken together, we propose that the mechanisms underlying persister formation should be more diverse than we thought previously, and Br-CA might be considered for the application in the infection treatment to overcome the persister problem if its toxicity could be proved to be acceptable in the future.

CA activates TRPA1 by covalently binding via Michael addition, leading to a Ca^2+^ influx [46]. Our previous study obtained similar results in which the stimulation of a transient Ca^2+^ efflux was involved in the CA-induced growth inhibition of *P*. *capsici* [20]. These studies imply that the potential of α, β-unsaturated bond undergoing Michael addition is directly associated with its antifungal activity. Br-CA has a very active α, β-unsaturated bond to allow for Michael addition [38], which seemed to correspond to its highest bactericidal activity. Exceptionally, α-methylcinnamaldehyde with an α, β-unsaturated bond did not display any bactericidal activity at any given concentrations, whereas phenylpropyl aldehyde without an α, β-unsaturated bond exerted bactericidal activity. This indicated that the bactericidal activity of CA derivatives was probably independent of Michael addition. In our present study, neither Thio (hydroxyl-radical scavenger) nor DPTA (Fe^3+^ chelator to block the hydroxyl-radical) affected the bactericidal efficiency of Br-CA against *E*. *coli*, which suggested a novel bactericidal mechanism of Br-CA independent of ROS. Taken together, our results indicated that Br-CA had efficient bactericidal activity with mechanisms different from common antibiotics, which implemented plant resources (e.g. CA and Br-CA) to be developed to tackle the antibiotics resistance crisis.
